# Occupational Exposure of Healthcare Workers During Drug Reconstitution and Handling Tasks: A Multicenter Assessment at Dutch Hospital Wards

**DOI:** 10.1097/JOM.0000000000003401

**Published:** 2025-04-18

**Authors:** Roland B. van den Berg, Percival Stubbs, Johanna R.C. Boumans-d’Onofrio, Christina W. Swart, Femke B. Steenstra, Paulien Oosterveld, Shiwai Ng, Marjolein Klous, Oscar Breukels, Eelco Kuijpers, Mirjam Crul

**Affiliations:** From the Department of Clinical Pharmacology and Pharmacy, Amsterdam UMC, location Vrije Universiteit, Amsterdam, the Netherlands (R.B.v.d.B., M.C.); Department of Hospital Pharmacy, Haaglanden Medisch Centrum, the Hague, the Netherlands (R.B.v.d.B.); Department of Human Resources, Health Team, Haaglanden Medisch Centrum, the Hague, the Netherlands (P.S.); Department of Human Resources, Occupational Health, Safety & Environmental Service, Radboudumc, Nijmegen, the Netherlands (J.R.C. B.-d’O.); Department of Clinical Pharmacy, University Medical Centre, Utrecht University, Utrecht, the Netherlands (C.W.S.); Occupational Health Department, University Medical Center Groningen, Groningen, the Netherlands (F.B.S.); Department of Human Resources, Leiden University Medical Center, Leiden, the Netherlands (P.O.); Laboratory of the Dutch Pharmacists, Royal Dutch Pharmacists Association, the Hague, the Netherlands (S.N.); Department of Pharmacy and Pharmacology, the Netherlands Cancer Institute-Antoni van Leeuwenhoek Hospital, Amsterdam, the Netherlands (M.K.); Department of Hospital Pharmacy, Meander Medical Center, Amersfoort, the Netherlands (O.B.); and Netherlands Organization for Applied Scientific Research, Utrecht, the Netherlands (E.K.).

**Keywords:** drug reconstitution, inhaled drug particles, occupational exposure, occupational exposure limits

## Abstract

**Objective:**

This study evaluated healthcare workers’ occupational exposure to inhaled drugs during drug reconstitution and handling tasks, focusing on compliance with occupational exposure limits (OELs).

**Methods:**

Ten nurses and pharmacy technicians were monitored during an 8-hour shift to measure inhalable particle concentrations using a portable sampling device.

**Results:**

Inhalable particle concentrations ranged from <15 to 381 μg/m^3^ (arithmetic mean 83 μg/m^3^). Exposure to drugs in hazardous drug classes (HDCs) 1, 2, and 3 complied with OELs, while exposure to drugs in HDCs 4 and 5 exceeded OELs, highlighting the need for enhanced protective measures.

**Conclusions:**

No additional precautions are needed for drugs in HDCs 1, 2, and 3, but dust exhaust or biosafety cabinets are recommended for handling drugs in HDCs 4 and 5.

## Abstract 2

This study highlights the occupational exposure risks healthcare workers face during drug reconstitution and handling. While exposure to lower-risk drugs complies with safety limits, higher-risk drugs exceed these limits, underscoring the need for enhanced protective measures like dust exhaust or biosafety cabinets to ensure worker safety.

## Graphical abstract

**Figure:**
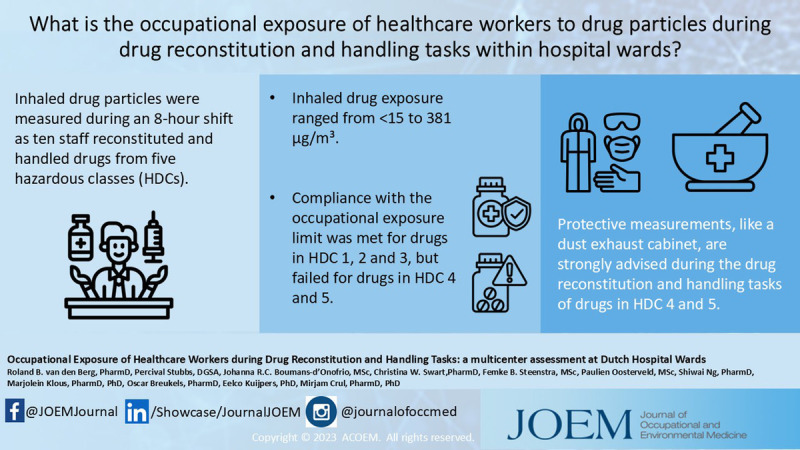

**LEARNING OUTCOMES**

- Occupational exposure during drug reconstitution and handling tasks complied with OELs for drugs in hazardous drug classes 1, 2, and 3 according to the SEG compliance test, but failed compliance for drugs in hazardous drug classes 4 and 5.
- Although compliance was observed for drugs in hazardous drug class 3, it is recommended that protective measures, such as dust exhaust cabinets or biosafety cabinets, limiting the duration of drug reconstitution or handling tasks and the use of personal protective equipment like FFP masks, be implemented.
- The use of dust exhaust cabinets or biosafety cabinets is strongly advised to reduce occupational exposure for drugs in hazardous drug classes 4 and 5.

Reconstitution or handling tasks of drugs are occasionally required for certain licensed drugs prior to their administration to patients. These tasks are typically executed within pharmacies, hospital wards and nursing facilities. Healthcare professionals may encounter both nonhazardous and hazardous substances during the reconstitution process preceding administration or dispensation. Therefore, the management of occupational exposure during pharmaceutical reconstitution is paramount for ensuring a safe working environment, as mandated by European Directives, and this includes the execution of a health risk assessment.^1–3^ Conducting a risk analysis based on potential occupational exposures during drug reconstitution or handling tasks enables the identification of necessary protective measures. Based on this risk analysis, employers are required to develop an occupational hygiene strategy and implement measures in accordance with national regulations. These measures may include the application of technical controls, the establishment of safe work processes, the provision of appropriate equipment and materials to prevent or significantly reduce the release of hazardous substances, or the use of personal protective equipment (PPE).^4^

The overall risk of occupational exposure to pharmaceutical agents depends on the hazardous nature of the drug and the extent of exposure to it.^5^ The extent of drug exposure encompasses factors such as the duration of exposure, the route of exposure (eg, oral, dermal, inhaled), the nature of the handling tasks, and the implementation of protective measures, including the use of Filtering Face Piece (FFP) masks, gloves, and dust exhaust cabinets. With the introduction of a new limit value system in 2007 due to revision of the European Commission Directive 2006/15/EC, private limit values became the standard.^6^ Consequently, employers have since been responsible for determining OELs and are increasingly accountable for ensuring workplace safety when handling hazardous substances. They must ensure that exposure levels remain within limits that do not endanger employee health. Given the impracticality of defining the risk for every drug in all potential scenarios, a risk matrix has been developed by Tielemans et al (2006) to categorize substance hazards.^7^ The risk matrix is utilized to assess occupational exposure to inhaled drugs within the defined limits during the compounding and reconstitution processes in pharmacies, as inhalation is the most likely route of exposure during these procedures. This risk matrix classifies drugs into five hazardous drug classes (HDCs), where class 1 represents the lowest occupational hazard and class 5 the highest. Commercially available drugs and pharmaceutically active compounds are assigned to one of these five HDCs based on their acute and chronic toxicity profiles, as indicated by hazard statements in accordance with CLP/REACH regulations.^8^ The toxicity of a drug product can be estimated based on the toxicity of its components, consisting of the active pharmaceutical ingredient (API) and excipients, using the Acute Toxicity Estimate (ATE) formula for mixtures and blends (see below).


$$\frac{100}{{\mathrm{ATE}}_{\mathrm{mix}}}=\sum_{n}\frac{C_{i}}{{\mathrm{ATE}}_{i}}$$

The ATE for each compound can be determined based on its concentration and the estimation of acute toxicity (ATEi), which is derived from median lethal dose (LD50) or median lethal concentration (LC50) values.^9^ Depending on the route of exposure—oral, dermal, or inhalation—and the calculated ATE_mix_, a drug product is categorized into one of five HDCs according to the classification scheme presented in Table S1, which aligns with the ATE values and criteria for acute toxicity hazard categories established by the World Health Organization (WHO).^9^ The OELs associated with these five HDCs, as outlined in the risk matrix by Tielemans et al (2006), are derived from the five hazard bands, which correspond to target airborne concentrations based on the R-phrases of the European Union classification system, as indicated by Brooke et al (1998).^10^ Consequently, each commercially available drug in the Netherlands has been categorized according to the risk matrix of Tielemans et al (2006).^7^

The health risk of pharmacy technicians during the reconstitution or handling tasks of drugs in pharmacies was investigated previously by Crul et al (2023). Their findings demonstrated that the mean particle concentrations varied from undetectable to 1.03 μg/m^3^ and 589.7 μg/m^3^, depending on the particle size. Further, they concluded that the occupational risk in pharmacies depends on the handling task and the HDC. Small to moderate health risks were found, depending on the type of drug reconstitution and handling task, when handling drugs for up to 8 hours per day in HDCs 1, 2, and 3. A dust exhaust cabinet is only advised for moderate risks. Additionally, no health risks were found when handling drugs for 15 minutes in HDCs 1 to 3. For drugs in HDCs 4 and 5, a dust exhaust cabinet in combination with a face mask with a ventilation valve is required, or a safety cabinet should be used, with an exception for the tasks of reconstituting powder in a vial and withdrawing fluid from a vial.^11^

As mentioned before, the reconstitution and handling of drugs are tasks not limited to pharmacies; they are also frequently performed by nurses and pharmacy technicians in hospital wards and nursing facilities. In most European countries, hazardous drugs with clearly defined carcinogenic, mutagenic, and/or reprotoxic (CMR) properties, such as cytotoxic drugs, are reconstituted in biohazard safety cabinets or isolators, adhering to standards to reduce and prevent occupational exposure.^12^ Furthermore, substantial knowledge exists regarding occupational exposure to CMR drugs and effective strategies to prevent such exposure.^13,14^ However, there is a lack of literature concerning the occupational risks to healthcare workers, such as nurses and pharmacy technicians, during the reconstitution and handling of other drugs in hospitals wards when no PPE or ventilation strategies are employed. Therefore, our objective is to assess the occupational exposure of healthcare workers during drug reconstitution and handling tasks within hospital wards.

## METHODS

### Outcome

The primary outcome measure of this observational study was the concentration of inhalable drug particle count (μg/m^3^) during the drug reconstitution and handling tasks over an 8-hour working shift. The secondary outcome was the concentration of respirable drug particle count (μg/m^3^) during the same drug reconstitution and handling tasks over an 8-hour working shift.

### Occupational Exposure Measurement Protocol and Sample Size

**FIGURE 1:**
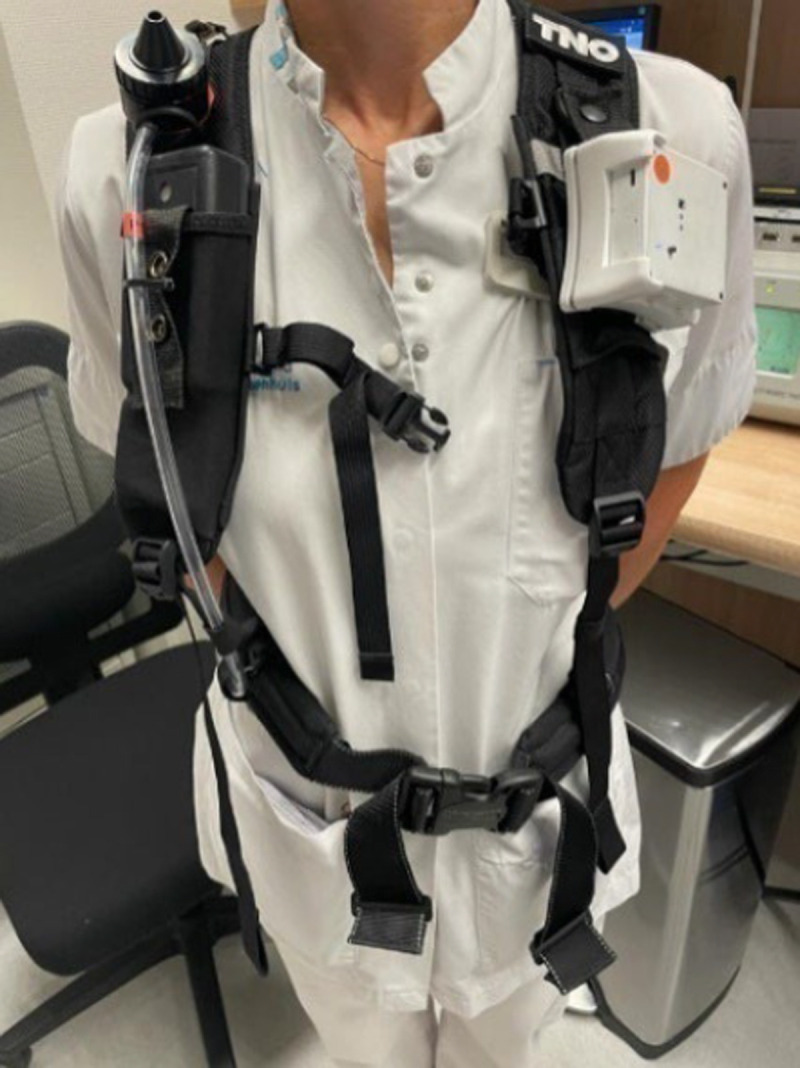
Vest worn by the participants with the GSP-10 sampling head, the air pump and EPHOR sensor attached.

On 5 days in four Dutch hospitals the occupational exposure to inhaled and respirable particles was measured during 8-hour working shifts of six nurses and four pharmacy technicians. The inhaled amount of particles was measured using the 8-hour working shift profile method, as specified by the European standard EN689, and these measurements were interpreted as representing inhaled drug particles.^15^ The inhalable particulate matter was measured continuously by the GSP-10 sampling head (GSA Messgerätebau GmbH, Ratingen, Germany) in combination with the GilAir wearable air pump (Sensidyne, LP, St. Petersburg, FL) and 37 mm Teflon filter (SKY Inc., Eighty Four, PA). The filter was weighted before and after the measurement period by using MX 5 Microbalance (Mettler Toledo, Geifensee, Zurich, Switzerland). The respirable particle matter was detected by using Plantower PMS7003 particulate matter sensor (VTEC Lasers & Sensors, Eindhoven, The Netherlands). This sensor measured the particles smaller than 1 μm (PM1), 2.5 μm (PM2.5), and 10 μm (PM10) each 20 seconds, where PM2.5 is the most suitable parameter of the respirable fraction. Each participant wore a vest to which the GSP-10 sampling head, the air pump and EPHOR sensor was attached. The sampling head was within a 20-cm inhalation zone of each participant (Fig. 1). Furthermore, to determine particle sizes ranging from 0.23 to 20 μm, an Aerodynamic Particle Sizer 3321 (APS)-spectrometer (TSI, Lynnwood, Washington, United States) was used. Particles smaller than 2.5 μm were categorized as the respirable fraction, and particles smaller than 10 μm were categorized as the inhalable fraction. Both inhalable and respirable particles were measured throughout the entire shift (ie, shift profile method).

In addition to personal measurements, stationary measurements were also conducted in the room where the reconstitution or handling tasks of drugs took place. The equipment used for the stationary measurements was identical to that used for the personal measurements. All reconstitution and handling tasks were performed in a non–Good Manufacturing Practice (GMP) classified closed medication room within a hospital ward. To create a worst-case scenario, the ventilation supply to the medication room was temporarily shut down during the 8-hour work shift. No PPE, besides nonsterile gloves that are used to prevent microbial contamination of the drug, were applied. The study was assessed and approved by institutional review board of the Medical Ethics Review Committee of the Dutch Organization for Applied Scientific Research (TNO). Reporting was in accordance with the STROBE guidelines (Supplementary Material, http://links.lww.com/JOM/B905).

### Drug Reconstitution and Handling Tasks

The occupational exposure was continuously measured over an 8-hour work shift for the participating healthcare workers. These healthcare workers were responsible for all drugs requiring reconstitution or handling tasks for the patients throughout the entire ward. The reconstitution and handling tasks for the drugs included the following:

- Task 1 Reconstituting powder in a vial. Here, the powder was dissolved in the vial and drawn up into a syringe.
- Task 2 Withdrawal of fluid from a vial.
- Task 3 Withdrawal of fluid from a glass ampoule with a filter needle.
- Task 4 Adding fluid to an infusion bag.
- Task 5 Crushing tablets in a tablet crusher and transferring the contents to a cup.
- Task 6 Opening a capsule and transferring the contents to a cup.
- Task 7 Disintegrating tablets or capsules in a syringe.
- Task 8 Preparing a powder for suspension
- Task 9 Manually splitting a tablet with a fault line.
- Task 10 Splitting tablets without a fault line using a tablet splitter.

A minimum of 20 reconstitution or handling tasks were required per 8-hour work shift to obtain a representative sample of the daily workload for each healthcare worker. Drug reconstitution and handling tasks were conducted based on the prescriptions of in-patients. Consequently, the type of tasks and the HDC of the drugs requiring reconstitution prior to administration varied, providing a realistic representation of daily drug reconstitution and handling processes, including exposure to different HDCs of drugs. If fewer than 20 tasks were performed because of a low number of drug prescriptions, simulated reconstitution or handling tasks were conducted to meet the required number of tasks. In the latter case, the prepared drug was discarded afterward and not used for patient care.

### Occupational Exposure Limits and Statistical Analysis

**FIGURE 2:**
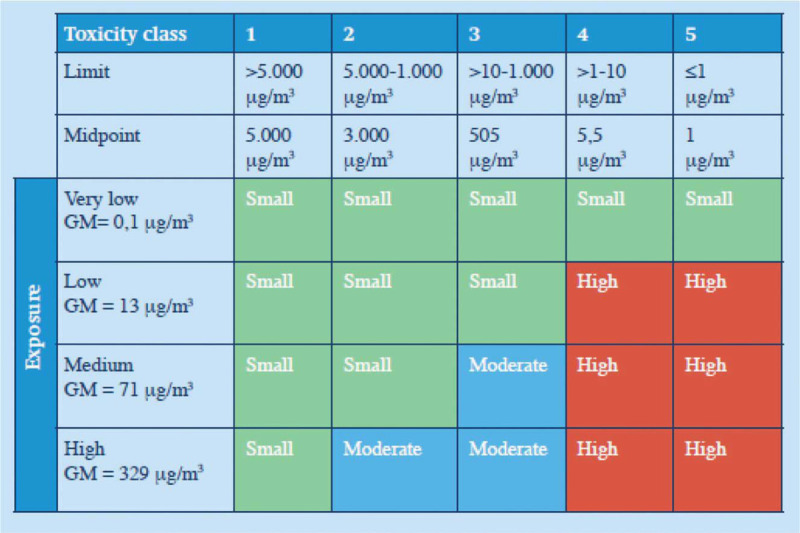
Risk matrix of inhaled drugs during compounding, reconstitution and handling tasks in pharmacies. The risk is stratified into three classes: small (green), moderate (blue), and high (red) and the exposure is described as the inhaled drug fraction. GM, geometric mean.^11^

In the absence of specific regulations and OELs for individual APIs or drug products in terms of an 8-hour time-weighted average, all APIs and drug products are classified into one of five HDCs within a national database. This classification is based on the chronic and acute toxicity of the API, as determined by H-statements, and is conducted in accordance with Regulation (EC) No. 1272/2008.^8^ As stated before, the OELs of inhaled drug particles were established for the five HDCs according to the risk matrix developed by Tielemans et al (2006).^7^ The OELs were defined at the midpoint of the respective limits, specifically: 5000 μg/m^3^ for class 1, 3000 μg/m^3^ for class 2, 505 μg/m^3^ for class 3, 5.5 μg/m^3^ for class 4, and 1.0 μg/m^3^ for class 5 (Fig. 2). In this study, each drug handled was classified into one of the five HDCs. Therefore, occupational exposure compliance was not determined for each individual drug and its associated OEL; instead, the total exposure to inhalable and respirable drug particles was assessed against the OELs of the five HDCs.

To evaluate compliance with the OELs, Similar Exposure Groups (SEG) compliance test was assessed in accordance with NEN-EN 689:2018.^15^ Compliance for normally distributed data can be determined using the following formula:


$$U_{r}=\frac{\mathrm{OEL}-\mathrm{AM}}{\mathrm{SD}}$$

The OEL can be assessed through the calculation of the arithmetic mean (AM) and the standard deviation (SD). Using these parameters, the test statistic *U*_r_ can be determined. If *U*_r_, the observed value of the test statistic, is greater than or equal to *U*_t_, the critical value of the test statistic, then the exposure is considered to comply with the OEL. The critical value *U*_t_ is contingent upon the number of exposure measurements. For ten measurements, the *U*_t_ is 2.005.

In contrast to inhaled drug particles, there are no established OELs for respirable drug particles, rendering the determination of occupational exposure to the respirable fraction impossible. Consequently, the assessment of occupational exposure to the respirable fraction serves only as an indicative measure, useful for determining whether the drug particles are primarily inhalable or respirable, and whether peaks in exposure are linked to specific drug reconstitution and handling tasks. Furthermore, it can help ascertain whether the observed occupational exposure is solely attributable to drug reconstitution and handling tasks. In addition to the personal measurements, the outcomes of stationary measurements of the inhaled and respirable fractions provide an indication of the occupational exposure for healthcare workers present in the room during drug reconstitution, but not directly involved in the reconstitution process.

## RESULTS

### Occupational Exposure of Inhaled and Respirable Drug Particles

A total of 10 personal and 5 stationary measurements were conducted over 5 days across four hospitals to determine the inhalable and respirable particle counts. The frequency of drug reconstitution or handling tasks totaled 338 and is presented in Table 1 for each type and participant. Drug reconstitution and handling tasks were performed using drugs from solely HDC 1, 2 and 3 (Table S2). The average inhalable fraction from the personal and stationary measurements ranged from <15 to 381 μg/m^3^ and from 17 to 119 with an arithmetic mean (AM) of 83 μg/m^3^ and 41 μg/m^3^, respectively (Table 2). Table 2 represents the number of drug reconstitutions and handlings tasks by each participant in relation to the average inhalable drug particles. The average respirable fraction (PM2.5) from the personal and stationary measurements varied from 0.02 to 1.29 μg/m^3^ and from 0.04 to 0.90 μg/m^3^ with an AM of 0.43 μg/m^3^ and 0.36 μg/m^3^, respectively (Table 3). Figure S1 to S10 (Supplementary Material, http://links.lww.com/JOM/B906) present the continuous measurements of the respirable fraction (PM1, PM2.5, and PM10) for all participants. Asterisks in the figures indicate the moments when drug reconstitution and handling took place.

**TABLE 1:** Overview of the Amount of Drug Reconstitutions and Handling Tasks per Type and Participant

Drug Reconstitution or Handling Task	Participant 1	Participant 2	Participant 3	Participant 4	Participant 5	Participant 6	Participant 7	Participant 8	Participant 9	Participant 10	Total
Hospital	A	A	B	B	C	C	D	D	C	C	
1	2	3	7	1	24	24	3	14	4	17	99
2			4		9	24		10	2	14	63
3	6	3	4	12	6	6	1	14		3	55
4	8	6	2	4	8	16	2	11	3	8	68
5	1	1	2	3			1	1	1	6	16
6	2					1		1		2	6
7											
8	1		1	3		2	4	2	7	8	28
9						1					1
10						1			1		2
Total	20	13	20	23	47	75	11	53	18	58	338

**TABLE 2:** Personal and Stationary Measurements of the Inhalable Fraction

Participant	Hospital	No. Drug Reconstitutions and Handling Tasks	Average Inhalable Fraction of Personal Measurement (μg/m^3^)	Average Inhalable Fraction of Stationary Measurement (μg/m^3^)
1	A	20	<15	119.3
2	A	13	381.4	
3	B	20	94.0	24.0
4	B	23	54.5	
5	C	47	44.2	22.7
6	C	75	49.2	
7	D	11	61.8	23.9
8	D	53	33.4	
9	C	18	44.6	17.4
10	C	58	49.0	

**TABLE 3:** Personal and Stationary Measurements of the Respirable Fraction

Participant	Hospital	Average Respirable Fraction of Personal Measurement (μg/m^3^)			Average Respirable Fraction of Stationary Measurement (μg/m^3^)		
		PM 1	PM 2.5	PM 10	PM 1	PM 2.5	PM 10
1	A	0.21	0.50	0.97	0.02	0.24	0.40
2	A	0.11	0.19	0.61			
3	B	0.65	0.90	1.69	0.29	0.90	1.21
4	B	0.65	1.29	2.15			
5	C	0.15	0.30	0.52	0.03	0.11	0.19
6	C	0.15	0.26	0.46			
7	D	0.09	0.23	0.45	0.23	0.48	0.82
8	D	0.28	0.54	0.93			
9	C	0.01	0.02	0.20	0.00	0.04	0.21
10	C	0.01	0.05	0.19			

### Compliance With OEL for Inhaled Drug Particles

Although the Shapiro-Wilk test indicated that the data do not follow a normal distribution (*P* = 2.086 × 10^−5^), they also do not follow a lognormal distribution (*P* = 0.001748). Based on the Q-Q plot, the data appear more normally distributed than lognormally distributed. Consequently, the SEG compliance test for normally distributed data was deemed more reliable for assessing compliance with the OELs. Using the arithmetic mean of the inhalable fraction, the SEG compliance test was conducted for drugs in the five HDCs. The calculated *U*_r_ values were 48.50 for HDC 1, 28.78 for HDC 2, 4.17 for HDC 3, −0.76 for HDC 4, and −0.81 for HDC 5. The *U*_r_ values for drugs in HDC 1 to 3 were above the *U*_t_ value of 2.005, indicating compliance with the OEL. However, occupational exposure during drug reconstitution and handling tasks involving drugs in HDCs 4 and 5 did not comply with the OEL, as the *U*_r_ values were lower than the *U*_t_ value of 2.005.

### Estimated Occupational Exposure and Risk Assessment for Drugs in HDC 3

Although occupational exposure during drug reconstitution and handling tasks for drugs in HDC 3 complies with the OEL, protective measures are recommended according to the risk matrix of Tielemans et al (2006) due to the moderate risk (occupational exposure with a geometric mean (GM) > 13 μg/m^3^).^7^ Protective measures may include the use of dust exhaust cabinets or biosafety cabinets, limiting the duration of drug reconstitution and handling tasks and the use of PPE, such as FFP masks. However, dust exhaust cabinets or biosafety cabinets are not always available or easily obtainable by healthcare organizations, whereas limiting the duration of reconstitution activities or PPE use are relatively easier to implement. Therefore, a strategy has been developed to estimate the inhaled drug exposure during drug reconstitution and handling tasks for drugs in HDC 3, considering both the duration and use of PPE (Table 4). By limiting the time spent on these tasks to below 1 hour per 8-hour working shift, using FFP masks, or employing a combination of both strategies, the estimated exposure can be reduced from moderate to low.

**TABLE 4:** Estimation of Inhalation Exposure (μg/m^3^) During the Drug Reconstitution and Handling Tasks of Drugs in HDC 3 (Excluding CMR and/or H334), in Conjunction With Duration and Use of Personal Protective Equipment

		Hours of Occupational Exposure per Day				
		1	2	4	6	8
Personal protective measures	None	10.4	20.8	41.5	62.3	83.0
	Face mask (FF)P1 (APF ≥ 4)	3.9	5.2	10.4	15.6	20.8
	Face mask (FF)P2 (APF ≥ 10)	1.6	2.1	4.2	6.2	8.3
	Face mask (FF)P3 (APF ≥ 20)	0.8	1.0	2.1	3.1	4.2

Numbers represent the estimated inhaled exposure to drug particles in μg/m^3^. The color indicates if the estimated inhaled exposure is low (green) or moderate (red), based on the limit of 13 μg/m^3^ where protective measures are advises.

APF, assigned protection factor; FFP, filtering face piece.

## DISCUSSION

Occupational exposure of healthcare workers to cytotoxic drugs, which are classified as hazardous medicinal products (HMPs) with CMR properties, has been documented when protective measures are not properly implemented or enforced.^16–24^ To mitigate this exposure, the European Commission has published guidelines for the safe management of HMPs in the workplace, which has been translated into national guidelines.^25,26^ However, there has been no investigation into the occupational exposure of healthcare workers to drugs in hospital wards, with the exception of cytotoxic drugs, and consequently, the safe management of these drugs may be insufficient. To the best of our knowledge, this study is the first to examine the occupational exposure of healthcare workers to non-CMR drugs during the reconstitution and handling of these drugs without protective measures in hospital wards.

### Interpretation and Implications of the Occupational Exposure of Inhaled Drug Particles

The results of this study concerning inhaled drug particles demonstrate that exposure to drugs in HDCs 1, 2, and 3 during drug reconstitution and handling tasks complies with the OEL based on the SEG compliance test. In contrast, drugs in HDCs 4 and 5 failed to meet the compliance criteria. Based on these findings, the use of a dust exhaust system or safety cabinet is strongly recommended for the reconstitution and handling of drugs in HDCs 4 and 5, while no additional protective measures are necessary for handling drugs in HDCs 1, 2, and 3. As previously mentioned, drug reconstitution and handling tasks were conducted in a nonventilated room without PPE, except for nonsterile gloves, to simulate a worst-case scenario. In practice, most medication rooms have a certain level of ventilation, and it is uncommon for a single nurse or pharmacy technician to perform all drug reconstitution and handling tasks for an entire ward throughout an 8-hour shift. This is further supported by the particles measured during stationary measurements, as the average concentration in stationary measurements is less than 50% of that observed in personal measurements. Therefore, our results may slightly overestimate actual exposure. Nevertheless, the exposure levels to drugs in HDCs 4 and 5 significantly exceed the OEL, indicating that the results of the SEG compliance test would likely remain unchanged for these two HDCs, even when room ventilation is considered without stratifying by reconstitution or handling task. Finally, the number of drug reconstitution and handling tasks does not appear to correlate with the concentration of the inhalable fraction (Table 2). However, the type of tasks varied significantly per participant, and research by Crul et al (2023) indicates that occupational exposure to inhaled drug particles could also be influenced by the type of task, in addition to protective measures.^11^

As mentioned earlier, in this study, each handled drug was classified into one of the five HDCs, and occupational exposure compliance was not determined for each individual drug and its associated OEL. Instead, the total exposure to inhalable and respirable drug particles was assessed against the OELs of the five HDCs. This approach was adopted because the amount of drug to be reconstituted or handled per HDC during a working shift is unknown in advance, as it depends on patient prescriptions and can vary daily or even from shift to shift. Furthermore, measuring cumulative occupational exposure over an 8-hour working shift and assessing compliance with the five OELs was considered a safer and more practical approach for employers compared to measuring occupational exposure for over 7000 registered drugs in the Dutch market and determining compliance for each drug.^27^ Additionally, it remains unclear whether occupational exposure to different drugs should be considered as individual exposures or whether exposure to multiple drugs during the same working shift should be aggregated. Therefore, we extrapolated the findings to simulate an employee’s exposure to inhaled drug particles during an 8-hour work shift for drugs within a single HDC. Subsequently, this extrapolation was extended to all HDCs over an 8-hour working shift to calculate a worst-case exposure scenario for each HDC, with the objective of ensuring a safe working environment under all conditions.

In contrast to our findings, Crul et al (2023) reported that the task reconstituting powder in a vial and withdrawing fluid from a vial with drugs in HDCs 4 and 5 could be performed without the use of dust exhaust cabinets or biosafety cabinets for up to 8 hours a day.^11^ A key difference between our study and that of Crul et al (2023) lies in the environment where the drug reconstitution and handling tasks were conducted, particularly regarding ventilation and room contamination, both of which we believe play a significant role. In our study, these tasks were carried out in a medication room without ventilation, whereas the standard ventilation rate is approximately 6 air changes per hour, which is used for multiple tasks beyond drug reconstitution and handling. In contrast, Crul et al (2023) conducted their tasks exclusively in pharmacies, with and without the use of dust exhaust cabinets, both situated in an environment with a ventilation rate of 16 air changes per hour. Furthermore, the stratification of occupational exposure by task was not conducted in this study, meaning that occupational exposure has not been determined per reconstitution or handling task. Therefore, we can only conclude whether drug reconstitution or handling tasks per HDC fall within acceptable limits, rather than evaluating them both per HDC and per individual drug reconstitution or handling task, as determined by Crul et al (2023) in a pharmacy setting. As a final point, the disparity in occupational exposure between the two studies may be attributed to differences in training and roles. Although all nurses and pharmacy technicians were trained and qualified to perform drug reconstitution and handling tasks in a non–GMP classified environment, it is well established that variations in the proper handling of drugs occur between pharmacies and wards.^28^ In conclusion, the observed discrepancies in exposure levels between the study by Crul et al (2023) and the present study are likely attributable to variations in ventilation and environmental standards in combination with the lack of task-specific stratification of occupational exposure.

In addition to compliance with the OEL, a risk matrix for compounding drugs has been developed by Tielemans et al (2006), which classifies the risk as low, medium, or high based on exposure levels that are categorized as very low (0.1 μg/m^3^), low (13 μg/m^3^), medium (71 μg/m^3^), or high (329 μg/m^3^) (Fig. 2).^7^ According to this risk matrix, the exposure level for drugs in HDCs 1 and 2 is considered low because the AM of exposure is 83 μg/m^3^, and a medium risk classification applies only to exposure levels of 329 μg/m^3^ or greater for drugs in HDC 2. The risk is classified as medium for drugs in HDC 3, as the AM of 83 μg/m^3^ exceeds the threshold of 71 μg/m^3^, which is the limit for a medium risk. Therefore, additional measures, such as, dust exhaust or biosafety cabinets, limiting the duration of drug reconstitution and handling tasks per day or using FFP masks, are recommended to reduce the risk to a low level. While room ventilation could theoretically play a role, the specific effects of ventilation on occupational exposure to inhaled drug particles remain unclear. Although these recommendations are not mandatory for compliance with the OEL, they could be implemented to reduce exposure and achieve a lower risk for certain drug reconstitution or handling tasks. The study by Tielemans et al (2006) also indicates that the handling of solid drug dosage forms, such as tablets or capsules, is associated with higher inhalation exposure compared to liquid or semisolid dosage forms, such as parenteral injections or infusions. In our study, the handling of solid drug dosage forms was slightly underrepresented compared to liquid or semisolid dosage forms. Therefore, occupational exposure may have been higher during the reconstitution of solid drug dosage forms. This highlights the need to implement measures for the reconstitution of drugs classified under HDC 3.

### Interpretation of the Occupational Exposure of Respirable Drug Particles

As previously mentioned, no OELs have been established for inhaled drug particles per HDC. Although the occupational exposure of the respirable fraction remains indicative due to the lack of an established OEL, these results may provide valuable insights in addition to those of the inhalable fraction. To begin with, continuous monitoring of the inhalable fraction could help determine whether peaks in exposure are linked to specific drug reconstitution and handling tasks. Figures S1, S2, and S5 to S10 (Supplementary Material, http://links.lww.com/JOM/B906) display various peak concentrations. However, the peaks in Figures S1, S2, S5, S6, S9, and S10 (Supplementary Material, http://links.lww.com/JOM/B906) show no correlation with any drug reconstitution or handling tasks, as indicated by the absence of asterisks around these peak concentrations. Only the peaks in Figures S7 and S8 (Supplementary Material, http://links.lww.com/JOM/B906) can be attributed to task 1 (reconstituting powder in a vial) and task 3 (withdrawing fluid from a glass ampoule using a filter needle). However, from earlier work by Crul et al (2023), it is known that reconstituting powder in a vial results in low occupational exposure; therefore, it is assumed that the observed peak in occupational exposure is due to task 3.^11^

Our continuous measurements revealed that observed occupational exposure is not solely attributable to drug reconstitution and handling tasks. In Figures S1 to S10 (http://links.lww.com/JOM/B906), overall occupational exposure is frequently comparable to periods when no drug reconstitution or handling tasks were being performed. This suggests that the respirable particles measured originated from sources other than drugs, and these contributed at least in part to the occupational exposure observed in this study. Potential causes include the lack of ventilation in the medication room (designed to simulate a worst-case scenario), other activities conducted in the medication room, outdoor air particles introduced during breaks, or nursing procedures and interventions. The particles were measured over the entire shift (shift profile method), rather than during drug reconstitution and handling tasks alone (task-specific profile method). This approach was preferred because of the limited time nurses or pharmacy technicians spent on these tasks per shift. Additionally, a lower detection limit would have been required if the task-specific profile method had been employed.

Finally, the majority of the particles are inhalable, as evidenced by the significantly higher exposure to PM10 particles compared to PM1 and PM2.5 particles (Table 3). This indicates that occupational exposure to inhalable particles, and subsequent compliance with the OEL, is of greater importance than that of the respirable fraction.

### Limitations

This study has several limitations. First, occupational exposure was measured across the entire working shift of nurses and pharmacy technicians, meaning particles were collected and measured during a variety of routine activities, such as nursing procedures and interventions. Furthermore, we were unable to determine whether the identified particles originated from reconstituted drugs or had an alternative source. As a result, the detected particles may not solely originate from drug reconstitution and handling tasks but could also come from other sources of occupational exposure, such as administering medication, drawing blood, or even from breaks taken for meals or smoking. Consequently, the occupational exposure to drug particles during drug reconstitution and handling may be overestimated.

Second, this study utilized the OEL for inhalable drugs classified into the five HDCs. However, the actual OEL may vary for individual drugs within the same HDC. Using an OEL for each drug would provide greater precision compared to an OEL assigned per HDC. Adopting this approach would require selecting a specific group of drugs and measuring occupational exposure exclusively during the reconstitution and handling tasks of those drugs. However, this would hinder the real-time assessment of occupational exposure over a standard 8-hour working shift for healthcare workers. Thus, the findings of this study provide a more representative evaluation of occupational exposure compared to the previously mentioned strategy.

Third, this study primarily focused on inhaled and respirable particles rather than the OEL for each API or drug product. Consequently, OELs for specific APIs or drug products were not assessed, which differs from the ISPE guideline Good Practice Guide: SMEPAC–Standardized Methodology for the Evaluation of Pharma Airborne Particle Emissions from Containment Systems, which recommends measuring API concentrations rather than particle levels.^29^ However, apart from this distinction, our methodology aligns with the SMEPAC approach. Furthermore, in accordance with Regulation (EC) No. 1272/2008 and information on the chronic and acute toxicity of APIs derived from H-statements, individual healthcare institutions could classify the drugs used within their organization and evaluate the associated risks based on the findings of this study. Nonetheless, future research should prioritize assessing exposure to specific APIs and drug products, rather than solely examining particulate matter to provide a more comprehensive evaluation of occupational exposure.

Fourth, no drug reconstitution or handling tasks involving drugs in HDC 4 and 5 were performed during this study. Therefore, the compliance assessment of occupational exposure to drugs in HDC 4 and 5 was based on the OEL for these categories combined with exposure data from drug reconstitution or handling tasks involving drugs in HDC 1, 2, and 3. This extrapolation introduces uncertainty regarding the actual exposure levels in HDC 4 and 5. Nevertheless, we do not anticipate that the 10 drug handling tasks included in this study would lead to significantly higher or lower particle levels in the healthcare workers’ work environments, because only particles were assessed and not the OELs per API or drug product.

Fifth, this study included ten measurements of 8-hour working shifts. However, to address the issue of variability and the relatively small number of measurements, the EN 689:2018 standard recommends using the SEG compliance test. The SEG compliance test allows for several measurements, with a minimum of six, to ensure the reliability of the results.^15^ Furthermore, we increased the number of measurements from six to ten, to enhance accuracy and provide more robust evidence.

Last, the frequency of drug reconstitution and handling tasks varied considerably among the 10 types of tasks (Table 2). Drug reconstitution or handling tasks involving types 1, 2, 3, and 4 occurred more frequently than those involving other categories. It is well-established that the type of task can influence occupational exposure.^11^ Although this study provides real-time monitoring of occupational exposure during drug reconstitution and handling, the type and quantity of tasks can vary substantially from day to day and across different hospital wards. As a result, occupational exposure levels might fluctuate if all 10 tasks were distributed proportionally. However, given the large difference between exposure levels and the OEL, the occupational exposure of the less frequently performed tasks would have to be disproportionately high to change the overall findings.

## CONCLUSIONS

In conclusion, the occupational exposure during drug reconstitution and handling tasks involving drugs in HDC 1, 2, and 3 complies with the OEL without the use of personal protective equipment (PPE), dust exhaust or biosafety cabinets, or specialized room ventilation. In contrast, such measures are advised for tasks involving drugs in HDC 4 and 5 to prevent exposure exceeding the OEL. Furthermore, the use of PPE or limiting task durations to less than 2 hours per shift is recommended for HDC 3 to reduce exposure, thereby mitigating the risk from a moderate to a low level. Future research should focus on establishing international OELs for each HDC, including the classification, enabling regulatory authorities to develop international guidelines, rather than allowing individual countries to set national guidelines based on the toxicity of the API, in accordance with Regulation (EC) No. 1272/2008.

## References

[1] European Parliament and the Council of the European Union. Council Directive 92/85/EEC of 19 October 1992 on the introduction of measures to encourage improvements in the safety and health at work of pregnant workers and workers who have recently given birth or are breastfeeding tenth individual directive. Available at: https://eur-lex.europa.eu/legal-content/EN/TXT/?uri=celex%3A31992L0085. 2019. Accessed May 20, 2024.

[2] European Parliament and the Council of the European Union. Council Directive 98/24/EC of 7 April 1998 on the protection of the health and safety of workers from the risks related to chemical agents at work (fourteenth individual Directive within the meaning of Article 16(1) of Directive 89/391/EEC) Available at: https://eur-lex.europa.eu/legal-content/EN/TXT/?uri=CELEX%3A31998L0024. 1998. Accessed May 20, 2024.

[3] European Parliament and the Council of the European Union. Directive 2004/37/EC of the European Parliament and of the Council of 29 April 2004 on the protection of workers from the risks related to exposure to carcinogens or mutagens at work (Sixth individual Directive within the meaning of Article 16(1) of Counc) Available at: https://eur-lex.europa.eu/legal-content/EN/TXT/?uri=celex%3A32004L0037. 2022. Accessed May 20, 2024.

[4] Government of the Netherlands. Arbeidsomstandighedenbesluit [Working Conditions Decree], Article 4.4. Available at: https://wetten.overheid.nl/BWBR0008498/2024-08-01/#Hoofdstuk4_Afdeling1_Paragraaf3_Artikel4.4. 2024. Accessed November 30, 2024.

[5] Gerding J Ng S Crauste-Manciet S. Occupational Safety and Health. In: le Brun P Crauste-Manicet S Krämer SJ Woerdenbag H, editors. *Practical Pharmaceutics*. Switzerland: Springer International Publishing; 2023. p. 605–662.

[6] European Parliament and the Council of the European Union. Commission Directive 2006/15/EC of 7 February 2006 establishing a second list of indicative occupational exposure limit values in implementation of Council Directive 98/24/EC and amending Directives 91/322/EEC and 2000/39/EC Available at: https://eur-lex.europa.eu/eli/dir/2006/15/oj/eng. 2006. Accessed February 23, 2025.

[7] Tielemans E le Feber M Groenewold M, . Risico’s tijdens het bereiden van geneesmiddelen in de apotheek (Dutch). TNO rapport 2006;V6288. Available at: https://www.rifas.nl/documentatie/rp_tno_v6288-2.pdf. 2006. Accessed May 20, 2024.

[8] European Parliament and the Council of the European Union. Regulation (EC) No 1272/2008 of the European Parliament and of the Council of 16 December 2008 on classification, labelling and packaging of substances and mixtures, amending and repealing Directives 67/548/EEC and 1999/45/EC, and amending Regulation (EC) Available at: https://eur-lex.europa.eu/legal-content/EN/TXT/?uri=celex%3A32008R1272. 2023. Accessed May 20, 2024.

[9] United Nations Economic Commission for Europe (UNECE). Globally harmonized system of classification and labelling of chemicals (GHS), 10th revised edition Available at: https://www.un-ilibrary.org/content/periodicals/24121576. 2023.

[10] Brooke IM. A UK scheme to help small firms control health risks from chemicals: toxicological considerations. *Ann Occup Hyg* 1998;42:377–390. 9738435 10.1016/s0003-4878(98)00050-7

[11] Crul M Breukels O Ng S Le Feber M Kuijpers E Smeets O. Limited health risks in performing drug reconstitution and handling tasks in pharmacies-results of an occupational risk assessment study. *J Occup Environ Med* 2023;65:e204–e210. 36728178 10.1097/JOM.0000000000002781 PMC10090273

[12] European Society of Oncology Pharmacy (ESOP). *Quapos: Quality Standard for the Oncology Pharmacy Service*. 6th ed. Available at: https://esop.li/wp-content/uploads/2020/01/QuapoS_english-6.pdf. 2018: Accessed May 20, 2024.

[13] Crul M Simons-Sanders K. Carry-over of antineoplastic drug contamination in Dutch hospital pharmacies. *J Oncol Pharm Pract* 2018;24:483–489. 28454501 10.1177/1078155217704990

[14] Crul M Hilhorst S Breukels O Bouman-d’Onofrio JRC Stubbs P van Rooij JG. Occupational exposure of pharmacy technicians and cleaning staff to cytotoxic drugs in Dutch hospitals. *J Occup Environ Hyg* 2020;17:343–352. 32633703 10.1080/15459624.2020.1776299

[15] Nederlands Normalisatie Instituut (NEN). NEN-EN 689: 2018. Workplace exposure - Measurement of exposure by inhalation to chemical agents - Strategy for testing compliance with occupational exposure limit values. Available at: https://www.nen.nl/nen-en-689-2018-en-246680. 2018. Accessed May 22, 2024.

[16] Connor TH Anderson RW Sessink PJ Broadfield L Power LA. Surface contamination with antineoplastic agents in six cancer treatment centers in Canada and the United States. *Am J Health Syst Pharm* 1999;56:1427–1432. 10428450 10.1093/ajhp/56.14.1427

[17] Connor TH McDiarmid MA. Preventing occupational exposures to antineoplastic drugs in health care settings. *CA Cancer J Clin* 2006;56:354–365. 17135692 10.3322/canjclin.56.6.354

[18] Fransman W Peelen S Hilhorst S Roeleveld N Heederik D Kromhout H. A pooled analysis to study trends in exposure to antineoplastic drugs among nurses. *Ann Occup Hyg* 2007;51:231–239. 17337460 10.1093/annhyg/mel081

[19] Fransman W Vermeulen R Kromhout H. Occupational dermal exposure to cyclophosphamide in Dutch hospitals: a pilot study. *Ann Occup Hyg* 2004;48:237–244. 15059800 10.1093/annhyg/meh017

[20] Hedmer M Tinnerberg H Axmon A Jönsson BAG. Environmental and biological monitoring of antineoplastic drugs in four workplaces in a Swedish hospital. *Int Arch Occup Environ Health* 2008;81:899–911. 18066576 10.1007/s00420-007-0284-y

[21] Castiglia L Miraglia N Pieri M, . Evaluation of occupational exposure to antiblastic drugs in an Italian hospital oncological department. *J Occup Health* 2008;50:48–56. 18285644 10.1539/joh.50.48

[22] Rioufol C Ranchon F Schwiertz V, . Administration of anticancer drugs: exposure in hospital nurses. *Clin Ther* 2014;36:401–407. 24612942 10.1016/j.clinthera.2014.01.016

[23] Hon C-Y Teschke K Chua P Venners S Nakashima L. Occupational exposure to antineoplastic drugs: identification of job categories potentially exposed throughout the hospital medication system. *Saf Health Work* 2011;2:273–281. 22953211 10.5491/SHAW.2011.2.3.273 PMC3430902

[24] Fransman W Vermeulen R Kromhout H. Dermal exposure to cyclophosphamide in hospitals during preparation, nursing and cleaning activities. *Int Arch Occup Environ Health* 2005;78:403–412. 15887018 10.1007/s00420-004-0595-1

[25] European Union. Guidance for the safe management of hazardous medicinal products at work Available at: https://op.europa.eu/en/publication-detail/-/publication/ee1e6d15-4095-11ee-952f-01aa75ed71a1/language-en. 2023. Accessed August 21, 2024.

[26] Nederlandse Federatie van Universitair Medische Centra (NFU). Veilig werken met geneesmiddelen [Safe working with medicines] Available at: https://www.dokterhoe.nl/risicos/veilig-werken-met-geneesmiddelen/. Accessed November 30, 2024.

[27] van Riet-Nales DA de Jager KE Schobben AFAM Egberts TCG Rademaker CMA. The availability and age-appropriateness of medicines authorized for children in the Netherlands. *Br J Clin Pharmacol* 2011;72:465–473. 21477143 10.1111/j.1365-2125.2011.03982.x PMC3175516

[28] Crul M Polidori C Paolucci D, . Centralization and automation of non-toxic drug reconstitution in the pharmacy: a strengths, weaknesses, opportunities, and threats analysis. *Int J Pharm Pract* 2024;32:97–99. 37897401 10.1093/ijpp/riad070

[29] International Society for Pharmaceutical Engineering (ISPE). Good Practice Guide: SMEPAC - Standardized Methodology for the Evaluation of Pharma Airborne Particle Emissions from Containment Systems. *2024*. Available at: https://ispe.org/publications/guidance-documents/good-practice-guide-smepac-standardized-methodology-evaluation-pharma-airborne-particle. Accessed February 21, 2025.

